# G Protein-Coupled Estrogen Receptor, GPER1, Offers a Novel Target for the Treatment of Digestive Diseases

**DOI:** 10.3389/fendo.2020.578536

**Published:** 2020-11-12

**Authors:** Chelsea DeLeon, David Q.-H. Wang, Christopher K. Arnatt

**Affiliations:** ^1^ Department of Chemistry, Saint Louis University, St. Louis, MO, United States; ^2^ Department of Medicine and Genetics, Division of Gastroenterology and Liver Diseases, Marion Bessin Liver Research Center, Einstein-Mount Sinai Diabetes Research Center, Albert Einstein College of Medicine, Bronx, NY, United States

**Keywords:** biliary sludge, bile salts, cholesterol gallstone disease, estrogen, estrogen receptors, gallbladder hypomotility, GPER1, GPER1 antagonists

## Abstract

There are gender differences between men and women in many physiological functions and diseases, which indicates that female sex hormones may be important. Traditionally, estrogen exerts its biological activities by activating two classical nuclear estrogen receptors, ESR1 and ESR2. However, the roles of estrogen in the regulation of physiological functions and the pathogenesis of diseases become more complicated with the identification of the G protein-coupled estrogen receptor (GPER1). Although many GPER1-specific ligands have been developed, the therapeutic mechanisms of exclusively targeting GPER1 are not yet well understood. Translational applications and clinical trial efforts for the identified GPER1 ligands have been focused primarily on the reproductive, cardiovascular, nervous, endocrine, and immune systems. More recently, research found that GPER1 may play an important role in regulating the digestive system. Cholesterol gallstone disease, a major biliary disease, has a higher prevalence in women than in men worldwide. Emerging evidence implies that GPER1 could play an important role, independent of the classical ESR1, in the pathophysiology of cholesterol gallstones in women. This review discusses the complex signaling pathways of three estrogen receptors, highlights the development of GPER1-specific ligands, and summarizes the latest advances in the role of GPER1 in the pathogenesis of gallstone formation.

## Introduction

The prevalence of digestive disease ranges from 10 to 27.8% in the United States (1, 2). Some common chronic digestive diseases include gallstone disease, nonalcoholic fatty liver disease, alcoholic liver disease, gastroesophageal reflux disease, irritable bowel syndrome, inflammatory bowel disease, gastric cancer, pancreatic cancer, and colon cancer. Many digestive disorders exhibit a distinct gender difference in prevalence between women and men (3–5), suggesting that sex hormones are important. Over the past decades, many basic research and clinical investigations have been focused largely on the roles of estrogen, through two classical nuclear estrogen receptors, ESR1 and ESR2 (also called ERα and ERβ), in the regulation of physiological functions and the pathophysiology of diseases such as cardiovascular, kidney, nervous, reproductive, endocrine, and gastrointestinal disorders. However, the discovery of a new estrogen receptor called the G protein-coupled estrogen receptor (GPER1) has made it more complicated to investigate the roles of estrogen in the pathogenesis of numerous diseases because estrogen can produce its biological activities through one of the three nuclear receptor signaling pathways, or a combination of two, or all three. This review discusses the latest advances in the signaling pathways of three estrogen receptors, the development of GPER1-specific ligands, and the roles of GPER1 and its ligands in the pathogenesis of cholesterol gallstone disease.

## Complex Signaling Pathways of Three Estrogen Receptors

**Figure 1:**
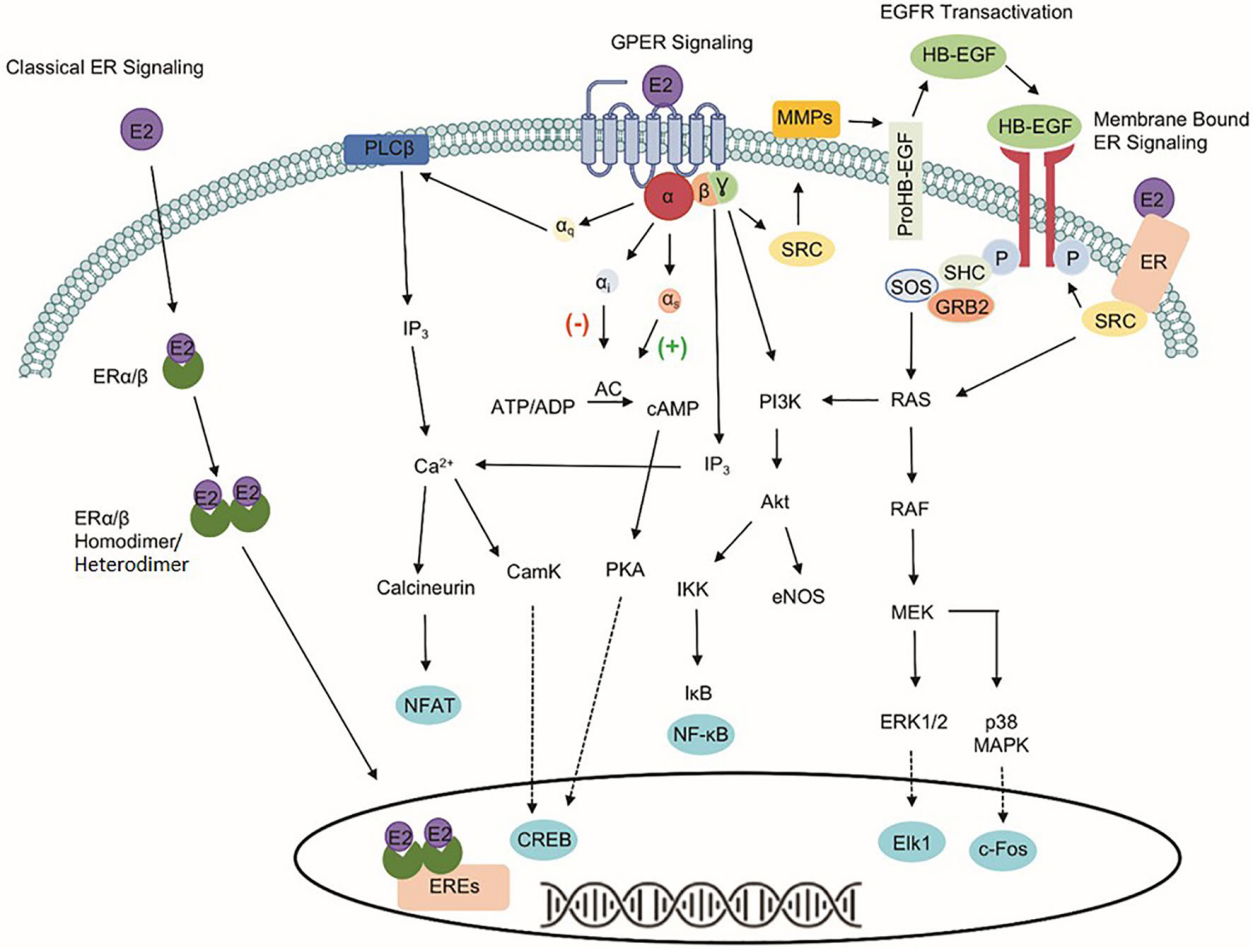
Signaling pathways of three estrogen receptors. The classical estrogen receptors, ESR1 and ESR2, primarily exist within the cytoplasm and nucleus, as well as interact with estrogen response elements (EREs) after dimerization to drive genomic signaling. Unlike the nuclear estrogen receptors, GPER1 signaling pathway occurs through various second messengers. Phospholipase C Beta (PLCβ), inositol triphosphate (IP_3_), nuclear factor of activated T-cells (NFAT), calcium/calmodulin-dependent protein kinase (CamK), cAMP response element-binding protein (CREB), adenylate cyclase (AC), protein kinase A (PKA), phosphoinositide 3-kinase (PI3K), protein kinase B (Akt), IκB kinase (IKK), nuclear factor kappa-light-chain-enhancer of activated B cells (NF-κB), endothelial nitric oxide synthase (eNOS), non-receptor tyrosine kinase (SRC), matrix metallopeptidases (MMPs), heparin-binding EGF-like growth factor (HB-EGF), son of sevenless (SOS), Src homology 2 domain-containing transforming protein (SHC), growth factor receptor-bound protein 2 (GRB2), RAS protein (RAS), RAF kinase (RAF), mitogen-activated protein kinase kinase (MEK), extracellular signal-regulated kinases 1/2 (ERK 1/2), Elk-1 transcription factor (Elk1), p38 mitogen-activated protein kinase (p38 MAPK), and c-Fos transcription factor (c-Fos).

The identification of three estrogen receptors has implied that estrogen-stimulated receptor signaling is more complex than initially realized (**Figure 1**). The naturally occurring estrogens are 17β-estradiol (E2), estrone, and estriol, and all of them are C_18_ steroids. Cellular response to E2 can occur through the activation of the nuclear estrogen receptors, ESR1, and ESR2. The classical ER signaling through the ERs involves the binding of estrogen, receptor dimerization, and subsequent association of coactivator proteins that guide the dimerized ER subunit to estrogen response elements (EREs) that drive transcriptional activity (6, 7). In addition, variants or single nucleotide polymorphisms (SNPs) in the *ESR1* and the *ESR2* genes increase the complexity and diversity of E2-mediated signaling transduction.

The identification of GPER1, a 375-amino acid protein known previously as GPR30, makes the well-known ER signaling pathways more complicated. Unlike the classical nuclear estrogen receptors, GPER1 signaling occurs through various second messengers (8–12). Specifically, GPER1 has been shown to activate ERK1/2 phosphorylation through G_β,γ_-dependent transactivation of epidermal growth factor receptor (EGFR), cAMP, calcium mobilization, and protein/lipid kinases (i.e., PKC and PKA) (8, 13–17). Interaction with these signaling pathways influences protein expression, apoptosis, cell proliferation, cell migration, and growth. Despite differences in signaling capabilities, ESR1, ESR2, and GPER1 are expressed ubiquitously throughout the human body and the variability of response in different tissues highlights the importance of understanding the druggability of each target separately due to the downstream signaling events that differ between the proteins (18).

## Current Ligands for the Modulation of GPER1 Signaling

**Figure 2:**
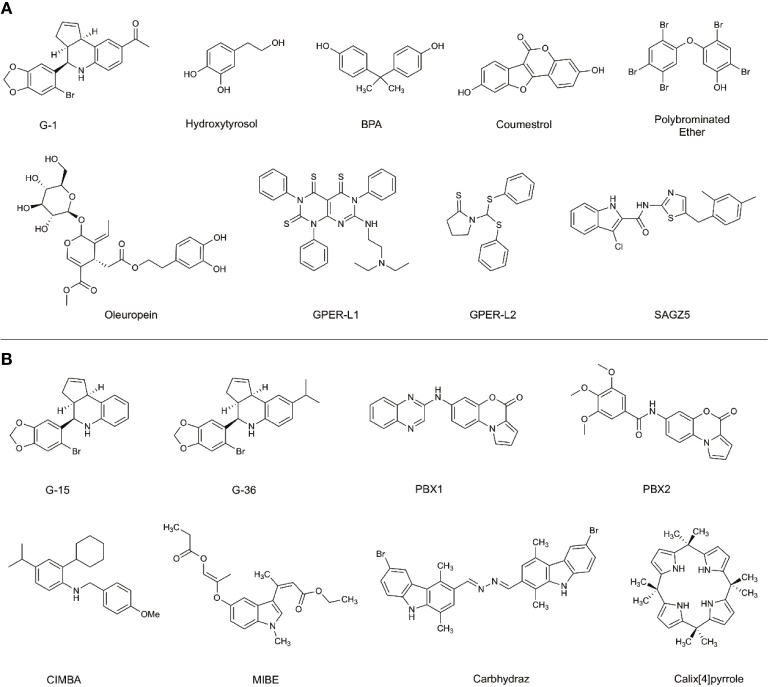
Selective and non-selective GPER1 agonists **(A)** and antagonists **(B)**. **(A)** Various GPER1 agonists have been identified. This includes non-selective natural products like hydroxytyrosol and oleuropein, phytoestrogens such as coumestrol, as well as endocrine-disrupting compounds like BPA. Various synthetic GPER1 agonists have been identified. A series of polybrominated ethers have been identified; however, these compounds likely do not exhibit specificity for GPER1. Several GPER1-specific agonists have been identified. These compounds include G-1, GPER-L1, GPER-L2, and SAGZ5. **(B)** Currently, there are no known naturally occurring GPER1 antagonists. Modifications were made to the tetrahydroquinoline scaffold of G-1 to create G-15 and G-36. These alterations modified the activity of the compounds to antagonists. Since the identification of G-15 and G-36, there have been a limited number of GPER1-specific antagonists identified. These include PBX1, PBX2, CIMBA, carbhydraz, and calix[4]pyrrole. MIBE has been identified as an antagonist for GPER1 and ESR1. In certain circumstances, there may be a therapeutic benefit in jointly targeting GPER1 and ESR1.

The effects of preferentially targeting GPER1 are not fully understood; therefore, there has been an increased research effort into the development of novel ligands to modulate GPER1 activity. Before the identification of GPER1-specific ligands, the antiestrogens, tamoxifen and fulvestrant, were shown to interact with GPER1 (19). While tamoxifen and fulvestrant block the ability of E2 to signal through ESR1 or ESR2, they also possess the ability to activate the GPER1 signaling pathway similarly to E2. The activity of antiestrogens at GPER1 highlights the cross-reactivity of estrogenic ligands and the difficulty in developing GPER1-specific ligands. In addition to antiestrogens, various non-selective GPER1 agonists have been identified: these include natural products like hydroxytyrosol and oleuropein, as well as phytoestrogens, such as coumestrol, and the endocrine-disrupting compounds Bisphenol A (BPA) (**Figure 2**) (20–22). Additional studies have identified synthetic polybrominated diphenyl ethers (PBDEs) and hydroxylated PBDEs as potential GPER1 ligands; however, these compounds likely exhibit no selective activity (23).

A hallmark challenge in the identification and discovery of GPER1-specific ligands has been the difficulty in achieving a crystalized structure of the receptor. Presently, a crystallized structure of GPER1 does not exist. For this reason, the identification and optimization of ligands has relied upon large-library virtual screening techniques and homology modeling (24–27). Due to the cross-reactivity of estrogenic ligands, a limited number of GPER1-specific ligands have been identified. The current benchmark for GPER1-specific ligands were identified through virtual screening of 10,000 into a model of GPER1 based on 2D- and 3D-similarity approaches and GPER-privileged substructures (24). From the screening, a substituted dihydroquinoline was identified and named GPR30-specific compound 1, G-1 (24). Binding studies revealed no appreciable binding to ESR1 or ESR2 below 100 nM (24). Subsequent functional bioassays with GPER1-transfected COS-7 cell and G-1 showed that E2 and G-1 exhibit an increase in calcium mobilization at 1 nM; however, a closer analysis of the data suggests that the kinetic profile of the calcium mobilization differs between the compounds such that G-1 exhibits slow receptor occupancy and an asymptotic curve and E2 exhibits fast receptor occupancy with a quick peak in calcium release (24). Medicinal chemistry approaches to modify the dihydroquinoline of G-1 altered the pharmacological activity of the scaffold from an agonist to an antagonist (28). While the identified antagonist, G-15, inhibited G-1 activity at GPER1, off-target binding to ESR1 and activation of EREs persisted (28). The reduction of binding and ERE activation was accomplished with the addition of an isopropyl group to the scaffold to make G-36 (29). While the G-series has become the standard for GPER1 agonists and antagonists, the success with the compounds has been variable and may be related to the tissue-specific signaling events of GPER1 (30, 31).

Since the development of the G-series of ligands, a limited number of groups have published data on synthesized ligands for GPER1. Lappano et al. proposed two tricyclic tetrahydroquinolines, GPER-L1 and GPER-L2 (32). These compounds were shown to bind exclusively to GPER1 without significant ESR1 binding above 100 μM (**Figure 2**) (33). Previously, we identified a series of N-thiazol-2-yl-1H-indole-2-carboxamide derivatives as GPER1 agonists (30). These compounds exhibited a similar effect on breast cancer proliferation as reported in the literature in response to the GPER1-selective agonist, G-1 (**Figure 2**) (30). Based on that work and further computational modeling, we have since reported the first structure-activity relationship for GPER1 antagonists and discovered CIMBA (2-cyclohexyl-4-isopropyl-N-(4-methoxybenzyl)aniline) (34). In addition to our group, Maggiolini et al. developed two selective GPER1 antagonists (PBX1 and PBX2) based on a benzo[b]pyrrolo[1,2-d][1,4]oxazin-4-one scaffold (**Figure 2**) (35). Both PBX1 and PBX2 effectively blocked agonist-induced GPER1 activity without transcriptional activation of the classical ERs. Additional non-selective GPER1 ligands have also been described in the literature. Unlike the GPER1 antagonists identified by DeLeon et al. and Maggioloini et al., Lappano et al. identified MIBE (ethyl 3-[5-(2-ethoxycarbonyl-1-methylvinyloxy)-1-methyl-1H-indol-3-yl]but-2-enoate) and demonstrated that MIBE blocks agonist activity at both GPER1 and ESR1 (36, 37). In addition to MIBE novel ligands, such as calixpyrrole derivatives that include a cyclic structure and resemble a porphyrin ring system, have been proposed as GPER1 antagonists (**Figure 2**) (36, 37).

The limited number of available GPER1-specific ligands may be attributed to a lack of clarity in the localization as well as the complex pharmacology associated with GPER1. The localization and expression of GPER1 has been long debated. Numerous studies have shown that GPER1 is expressed both along the cell membrane surface as well as intracellularly within the endoplasmic reticulum and Golgi apparatus (38–40). After several decades, it is now recognized that even though GPER1 expression exists within the cell membrane, the expression level is substantially less than the subcellular expression (40). This has important implications for drug discovery in that GPER1 ligands may need to be lipophilic and able to cross the cell membrane to access the receptor. The data achieved relating to the pharmacology of the G-series has varied among groups and has posed challenges to defining G protein coupling (31, 40). Together, the localization and varied success with currently available probes substantiate the need for novel GPER1-specific ligands to better understand the pharmacology associated with GPER1 and the clinical implications for the receptor.

## Role of GPER1 in Cholesterol Gallstone Disease

Cholesterol gallstone disease is one of the most prevalent and costly digestive diseases in the United States, with at least 20 million Americans (12% of adults) being affected (41). Clinical and epidemiological investigations have demonstrated that women are twice as likely as men to form cholesterol gallstones in every population that has been studied (42). Oral contraceptives and conjugated estrogens significantly increase gallstone prevalence in premenopausal and postmenopausal women (43–53). Similar lithogenic effects are also found in men with prostate cancer during postoperative estrogen therapy (54–56). All these studies show that E2 is a critical risk factor for gallstone disease and a high predisposition to gallstones in women than in men is related to differences in how the liver metabolizes cholesterol in response to E2 (57). Although both ESR1 and ESR2 are expressed in the liver of mice and humans, ESR1 expression is approximately 50-fold higher compared to ESR2 expression (58). Despite these observations, the mechanism by which ESR1 plays a key role in mediating E2-induced lithogenic actions at a cellular and molecular level is not yet fully understood. Exciting results show that E2 enhances cholelithogenesis by increasing hepatic expression of ESR1 but not ESR2, and the lithogenic actions of E2 can be blocked completely by the antiestrogenic agent, ICI 182,780 (58). Furthermore, the ESR1-selective agonist propylpyrazole, but not the ESR2-selective agonist diarylpropionitrile, promotes hepatic cholesterol output, leading to cholesterol-supersaturated bile and gallstones (58). Similar to E2 treatment, tamoxifen significantly increased biliary cholesterol secretion and gallstone prevalence (58, 59). These results indicate that the hepatic ESR1, but not ESR2, plays a critical role in E2-induced gallstones in female mice. More importantly, ESR1 stimulated by E2 dramatically increases hepatic expression of sterol regulatory element-binding protein-2 (SREBP-2), activating SREBP-2-responsive genes in the cholesterol biosynthetic pathway (60). Thus, the E2-treated mice continue to synthesize cholesterol despite its excess availability from high dietary cholesterol, which reflects a loss in controlling the negative feedback regulation of cholesterol synthesis. As a result, more newly synthesized cholesterol determined by the estrogen-ESR1-SREBP-2 pathway is secreted into bile, leading to biliary cholesterol hypersecretion and enhancing the lithogenicity of bile (60).

More interestingly, the deletion of *Esr1* diminishes susceptibility to E2-induced gallstones by reducing hepatic cholesterol secretion and desaturating gallbladder bile; however, this cannot completely protect against gallstone formation in mice treated with high doses of E2 and fed the lithogenic diet (61). As found by a powerful genetic quantitative trait locus (QTL) analysis, *Gper1* is a new gallstone gene, *Lith18*, on chromosome 5 in mice (62–66). GPER1 activated by its agonist, G-1, enhances cholelithogenesis by deterring expression of cholesterol 7α-hydroxylase, the rate-limiting enzyme for the classical pathway of bile salt synthesis (67). These metabolic abnormalities greatly increase biliary cholesterol concentrations in company with hepatic hyposecretion of biliary bile salts, leading to cholesterol-supersaturated gallbladder bile and accelerating cholesterol crystallization (68). Moreover, E2 activates GPER1 and ESR1 toproduce liquid crystalline versus anhydrous crystalline metastable intermediates evolving to cholesterol monohydrate crystals from supersaturated bile (69). However, cholesterol crystallization is drastically retarded in *Gper1*/*Esr1* double knockout mice. This indicates that GPER1 produces a synergistic lithogenic action with ESR1 to enhance E2-induced gallstone formation.

Impaired gallbladder motility is often a distinctive clinical feature of pregnant women and subjects received high doses of E2, which promotes the formation of biliary sludge, the precursor of gallstones (70–75). Immunohistochemical studies find that GPER1 is expressed predominately in the epithelial cells of the gallbladder (69). By contrast, ESR1 is expressed mainly in the smooth muscle of the gallbladder (69). This suggests that GPER1 could impair gallbladder motility, working independently of ESR1, as both can cause sluggish gallbladder contractility from different mechanisms. Indeed, G-1 impairs gallbladder emptying through the GPER1 pathway in mice, leading to sluggish gallbladder motility and accelerating the development of biliary sludge in the early stage of gallstone formation (67).

More recently, exciting evidence shows that a novel, potent GPER1-selective antagonist, CIMBA, reduces the prevalence of E2-induced gallstones in a dose-dependent manner by impeding the GPER1 signaling pathway in female wild-type mice (76). However, gallstones can be completely prevented in E2-treated ESR1 knockout mice even on the lithogenic diet (76). These results are consistent with the findings that the deletion of either *Esr1* or *Gper1* significantly reduces the prevalence of E2-induced gallstones but could not abolish it completely.

Overall, these studies have established a novel concept that GPER1 is involved in E2-dependent lithogenic actions, working independently of ESR1, as both GPER1 and ESR1 can promote the formation of E2-induced gallstones through different pathways. Thus, both GPER1 and ESR1 are potential therapeutic targets for cholesterol gallstone disease, particularly in women and patients exposed to high levels of E2 (77).

## Conclusions and Future Directions

The similarity between estrogenic compounds poses significant challenges in the design of new, selective ligands due to the promiscuous binding of estrogenic compounds to different types of ERs and is a particular challenge for designing new compounds. While estrogen binding is frequently associated with the nuclear ERs, GPER1 has been recognized as a new ER. A frequently neglected aspect of ER signaling is the ability of E2 and estrogenic compounds to directly activate calcium channels, specifically L-type calcium channels and calcium-activated BK (big potassium) channels (78, 79). The activation of ion channels by estrogenic compounds adds another level of complexity to studying ER signaling pathways and the design of GPER1-specific compounds.

The signaling pathways of ERs are complex and multifaceted. For this reason, studies that aim to examine a singular ER signaling pathway should not neglect existence of the three ERs. The therapeutic implications of targeting multiple ER signaling pathways are not well understood; however, evidence exists that cross-reactivity may severely limit the application of certain therapeutics. For instance, even though selective estrogen receptor modulators (SERMs) exhibit antiestrogen effects at the classical ERs, the cross-reactivity and activation of GPER1 may contribute to therapeutic resistance, which renders the therapeutic ineffective (80, 81). This limitation has been observed with tamoxifen. Alternatively, there may be some therapeutic opportunities for cross-reactivity, specifically in the gallbladder. In this circumstance, previous evidence has shown that inhibition of ESR1 or GPER1 alone is not sufficient to completely prevent gallstone formation (49). In this instance, the cross-reactivity of a compound, such as MIBE (**Figure 2**), which acts as an antiestrogen at both ESR1 and GPER1 may be a useful tool. The identification of new agonists has largely occurred in breast cancer cell lines that endogenously express GPER1. The pharmacology associated with GPER1 may be tissue-specific since GPER1 is expressed ubiquitously throughout the body. The use of additional cell lines may lead to a greater number of potent and efficacious ligands.

In most areas of the digestive system, there are still opportunities for further understanding the impact of exclusively targeting GPER1 and understanding the potential pharmacological implications of targeting multiple ERs. While G-1 has served as a valuable tool for understanding the role of GPER1 in health and disease associated with the digestive system in animals, and development of further GPER1 agonists and antagonists will lead to potential therapeutics with greater activity, specificity, and solubility in water or oil. The role of GPER1 in cholesterol gallstone disease presented in this review highlights the potential importance of GPER1 in hepatobiliary diseases. Overall, the prevention of lithogensis *via* a GPER1 antagonist represents a novel treatment option for high-risk populations and may prove to be an adjunct therapy to nonsurgical gallstone treatments.

## Author Contributions

All authors equally contributed to writing this perspective. All authors contributed to the article and approved the submitted version.

## Funding

This work was supported in part by the start-up funds from Saint Louis University (to CKA), as well as by research grants DK106249, DK114516, and DK126369 (to DQ-HW), as well as P30 DK041296 (to Marion Bessin Liver Research Center), all from the National Institutes of Health (US Public Health Service).

## Conflict of Interest

The authors declare that the research was conducted in the absence of any commercial or financial relationships that could be construed as a potential conflict of interest.

## Abbreviations

E2, 17β-estradiol; ER, estrogen receptor; ERE, estrogen response element; ESR1, estrogen receptor α; ESR2, estrogen receptor β; 2-ME, 2-methoxy-estradiol; GPER1, G protein-coupled estrogen receptor; QTL, quantitative trait locus.
